# Protein Inhibitor of Activated STAT2 Restricts HCV Replication by Modulating Viral Proteins Degradation

**DOI:** 10.3390/v9100285

**Published:** 2017-09-30

**Authors:** Jing Guo, Dan Chen, Xiaoxiao Gao, Xue Hu, Yuan Zhou, Chunchen Wu, Yun Wang, Jizheng Chen, Rongjuan Pei, Xinwen Chen

**Affiliations:** 1 State Key Laboratory of Virology, Wuhan Institute of Virology, Chinese Academy of Sciences, Wuhan 430071, China; guojing122@hotmail.com (J.G.); DanChen1005@163.com (D.C.); gaoxiaoxiao520@126.com (X.G.); huxue@wh.iov.cn (X.H.); zhouyuan325@163.com (Y.Z.); wucc@wh.iov.cn (C.W.); wangyun@wh.iov.cn (Y.W.); chenjz@wh.iov.cn (J.C.); 2 University of Chinese Academy of Sciences, Beijing 100049, China

**Keywords:** Hepatitis C virus, PIAS2, protein stability

## Abstract

Hepatitis C virus (HCV) replication in cells is controlled by many host factors. In this report, we found that protein inhibitor of activated STAT2 (PIAS2), which is a small ubiquitin-like modifier (SUMO) E3 ligase, restricted HCV replication. During infection, HCV core, NS3 and NS5A protein expression, as well as the viral assembly and budding efficiency were enhanced when endogenous PIAS2 was knocked down, whereas exogenous PIAS2 expression decreased HCV core, NS3, and NS5A protein expression and the viral assembly and budding efficiency. PIAS2 did not influence the viral entry, RNA replication, and protein translation steps of the viral life cycle. When expressed together with SUMO1, PIAS2 reduced the HCV core, NS3 and NS5A protein levels expressed from individual plasmids through the proteasome pathway in a ubiquitin-independent manner; the stability of these proteins in the HCV infectious system was enhanced when PIAS2 was knocked down. Furthermore, we found that the core was SUMOylated at amino acid K78, and PIAS2 enhanced the SUMOylation level of the core.

## 1. Introduction

Hepatitis C virus (HCV) is one of the major causes of chronic liver disease and is an enveloped, positive-strand RNA virus that belongs to the *Flaviviridae* family [1]. After translation from genomic RNA, the HCV polyprotein is cleaved by host and viral proteases into ten viral proteins, including structural proteins (core, E1 and E2) and nonstructural proteins (p7, NS2, NS3, NS4A, NS4B, NS5A and NS5B) [2]. The core is the capsid protein and participates in virion particle formation and HCV pathogenesis. The nonstructural proteins form the replication complex and coordinate viral RNA replication. Among them, NS3 is a multifunctional protein with serine protease and RNA helicase activities, and NS5A interacts with other viral and cellular proteins and functions in viral replication and assembly. NS3, NS5A and the NS5B RNA-dependent RNA polymerase (RdRp) are targets for anti-viral drug development.

HCV manipulates a wide range of cellular responses to facilitate its replication. For example, intracellular membranes are rearranged to form so-called membranous web structures [3], and lipid droplet numbers are increased and accumulated [4]; these processes are required for HCV replication and assembly, respectively. Conversely, host cells have developed tactics to restrain viral replication. In addition to the innate immune response, which inhibits viral replication through interferon (IFN) production [5], several non-IFN-induced host factors, such as ficolin-2 [6], apolipoprotein B messenger RNA editing enzyme catalytic polypeptide-like 3G (APOBEC3G) [7], suppressor of actin 1 (SAC1) [8,9], Y-box-binding protein 1 (YB1) [10] and protein kinase D (PKD) [11], have been reported to restrict HCV replication at the steps of entry, replication, particle production, secretion and release in the HCV life cycle. Modulating the stability of viral proteins is another method to confine viral replication. For example, HCV infection activates the endoplasmic reticulum (ER)-associated degradation (ERAD) pathway, which subsequently targets E2 for ubiquitylation and proteasomal degradation [12]. The core [13,14], E2 [15], NS5A [16] and NS5B [17] proteins have all been reported to be ubiquitinated by different E3 ligases and thus targeted for proteasomal degradation. The NS5A protein has been reported to be recruited to the autophagy-lysosomal degradation pathway by shisa family member 5 (SCOTIN) [18].

Protein inhibitor of activated STAT2 (PIAS2) is a human, small ubiquitin-like modifier (SUMO) E3 ligase, and mediates the SUMO modification (SUMOylation) of many host and viral proteins, such as the NP protein of influenza A virus [19], immediate-early protein Rta of Epstein–Barr virus [20], capsid protein of Moloney murine leukemia virus [21], and E1 protein of papillomavirus [22]. Similar to ubiquitination, SUMOylation is a cascade process mediated by E1-activating enzyme, E2-conjugating enzyme and E3 ligating proteins [23,24]. A common feature of SUMOylation is the change in the molecular interactions of the SUMOylated proteins, which ultimately result in changes in protein activity, localization or stability [25]. Unsurprisingly, both enhanced and restricted effects of SUMOylation on viral replication have been reported due to the diverse fates of SUMOylated proteins. For example, stable SUMO expression inhibits vesicular stomatitis virus (VSV) infection by stabilizing the MxA protein [26], which is known to inhibit VSV primary transcription [27]. The SUMOylation of Dengue virus (DENV) NS5 increases the stability of the NS5 protein and enhances viral replication [28].

In this report, we found that PIAS2 restricted HCV replication at the protein expression, viral assembly and budding levels. Knockdown or overexpression of PIAS2 modulated the stability of the HCV core, NS3 and NS5A proteins. PIAS2 mediated degradation of the HCV core, NS3 and NS5A proteins through the proteasome pathway, which required the SUMO E3 ligase function of PIAS2. Finally, the core protein was identified as SUMOylated at amino acid K78.

## 2. Materials and Methods

### 2.1. Cell Lines and Virus

Huh7 cells and human embryonic kidney HEK-293T cells were maintained in Dulbecco’s modified Eagle’s medium (DMEM) (Gibco, New York, NY, USA) containing 10% fetal bovine serum (FBS) (Invitrogen, Grand Island, NY, USA). The subgenomic HCV replicon cell line (Con1) containing subgenomic genotype 1b HCV was grown in the same medium supplemented with 0.5 mg/mL G418 [29]. The HCV J399EM strain with an insertion of the enhanced green fluorescent protein (EGFP) in the NS5A region of the JFH1 backbone was described previously [30].

### 2.2. Plasmids

pHA-PIAS2 was constructed by cloning human PIAS2 (GenBank accession number NM_004671.3) cDNA into pXJ40-HA. The plasmids expressing the FLAG-tagged HCV core, E1, E2, NS2, NS3, NS4A, NS4B, NS5A and NS5B proteins of genotype 2a (JFH1; GenBank accession no AB047639), pNL4.3.lucR^−^E^−^, pcDNA3.1-E1E2 and pHCV-internal ribosome entry site (IRES) were described previously [29,31]. pUb was constructed by cloning human ubiquitin cDNA into pcDNA3.1. The following plasmids were kindly provided by different groups: human SUMO1, SUMO2/3 expression plasmids from Bing Sun (Institute Pasteur of Shanghai) [19], pSENP1 (SUMO1/sentrin specific peptidase 1) from Jinke Cheng (Shanghai Jiao Tong University), and pHA-PA28γ (proteasome activator 28-γ) from Xiaotao Li (East China Normal University). The plasmids expressing the ubiquitin protein ligase E3A (E6AP), heat shock protein 90 (Hsp90), S-phase kinase-associated protein 2 (SKP2), were provided by the Microorganisms & Viruses Culture Collection Center, Wuhan Institute of Virology, CAS.

### 2.3. RNA Interference (RNAi)

The small interfering RNA (siRNA) specific for human PIAS2 (5′-AAGATACTA AGCCCACATTTG-3′) [32] was synthesized by GenePharma, Suzhou, China. The siRNAs were transfected twice, as described previously [31].

### 2.4. RNA Extraction and Real-Time RT-PCR

Total RNA was isolated with the TRIzol or TRIzol LS reagent (Invitrogen, Carlsbad, California, USA), and real-time RT-PCR was performed using a QuantiFast SYBR green RT-PCR kit (Qiagen, Hilden, Germany) as previously described [31]. The following primers were used for the real-time RT-PCR: PIAS2 sense: 5′-CTCATCAAGCCCACGAGTTTAG-3′ and antisense: 5′-CCAGGCAAAGTCTCAACTGAA-3′; HCV sense: 5′-ATCACTCCCCTGTGAGGAACT-3′ and antisense: 5′-GCGGGTTGATCCAAGAAAGG-3′; the primers for actin were previously described [33].

### 2.5. Viral *Titration,* Calculation of Virus Assembly, Budding Efficiency and Specific Infectivity

The infectious HCV titers in culture supernatants were determined by the endpoint dilution assay described by Lindenbach et al. [34]. To determine the intracellular virus titer, cells were washed with phosphate buffer saline (PBS) 3 times and collected into 1.5 mL tubes, lysed by four rounds of freeze–thaw using temperatures of −80 °C and 37 °C. After a centrifugation at 4000 rpm for 5 min, the supernatant was collected and the virus titer determined. The virus assembly efficiency, budding efficiency and specific infectivity were defined as, the ratio of the supernatant viral RNA copy number to that in the cells, the ratio of the supernatant viral titer to that in the cell lysate [35,36] and the ratio of supernatant viral titer to viral RNA copy number [37].

### 2.6. HCVpp Transduction and HCV Subgenomic RNA Electroporation

HCVpp was generated as previously described [38]. HCV subgenomic RNA was in vitro synthesized following the MEGAscript T7 Kit (Ambion, Austin, TX, USA) protocol. For subgenomic RNA electroporation, Huh7 cells were resuspended in Cytomix (120 mM KCl; 0.15 mM CaCl_2_; 10 mM K_2_HPO_4_/KH_2_PO_4_ (pH 7.6); 25 mM Hepes; 2 mM EGTA; 5 mM MgCl_2_) at a density of 1.0 × 10^7^ cells/mL. Cell suspension (400 μL) was then mixed with in vitro-transcribed HCV subgenomic RNA (10 μg) and pulsed (270 V, 975 μF) using a Gene Pulser Xcell™ apparatus (Bio-Rad, Hercules, California, USA, cat. no. 165-2660) [39].

### 2.7. Immunoprecipitation (IP) and Western Blotting

IP was performed as previously described [29]. Proteins were separated by 12% polyacrylamide gel electrophoresis (SDS-PAGE) and transferred to a nitrocellulose membrane (Merck Millipore, Darmstadt, Germany). The membrane was blocked in Tris-buffered saline with Tween 20 (TBST) containing 5% skim milk and incubated with specific primary antibodies, including anti-core (catalog no. ab2740; Abcam, Cambridge, UK), anti-NS3 (catalog no. ab65407; Abcam Cambridge, UK), anti-NS4B (catalog no. ab24283; Abcam, Cambridge, UK), anti-NS5B (catalog no. 3E5; BioFront, Tallahassee, FL, USA), anti-GFP (catalog no. M20004; Abcam, Cambridge, UK), anti-FLAG (catalog no. F1084; Sigma-Aldrich, St Louis, Mo, USA), anti-HA (catalog no. H9658; Sigma-Aldrich, St Louis, Mo, USA), anti-V5 (catalog no. R960-CUS; Invitrogen, Grand Island, NY, USA), anti-SUMO1 (catalog no. sc-5308; Santa Cruz, California, USA), anti-ubiquitin (catalog no. ab7780; Abcam, Cambridge, UK) and anti-β-actin (catalog no. sc-47778; Santa Cruz, California, USA). The secondary antibodies were horseradish peroxidase (HRP)-conjugated goat anti-mouse/rabbit IgG antibodies (Invitrogen, Grand Island, NY, USA). The membrane-bound antibodies were detected with the Super Signal-Femto chemiluminescent substrate (Pierce, Waltham, MA, USA).

### 2.8. SUMOylation Assay

To determine whether the HCV core protein was SUMOylated, 293T cells were transfected with the indicated plasmids. After 48 h of transfection, the cells were washed with PBS and lysed in immunoprecitipation (IP) buffer plus 20 mM *N*-ethylmaleimide (NEM, Sigma) for 30 min. The lysates were centrifuged at 12,000× *g* for 10 min at 4 °C. The supernatant was immunoprecipitated with primary antibodies and incubated with protein A/G agarose (Millipore) for 4 h at 4 °C. The beads were washed with IP buffer and then boiled in loading buffer. The samples were subjected to SDS-PAGE gradient gels (8–15%) and western blotting analysis.

### 2.9. Protein Degradation Assay

HCV protein degradation was detected via cycloheximide (CHX) chase analysis. After 0, 2, 4, 8 h pretreatment with CHX (100 μg/mL), the cells were collected and assayed by Western blotting.

### 2.10. Statistical Analysis

The data were analyzed using a two-tailed unpaired *t* test. P values were calculated and reported as significant when *p* ≤ 0.05 (*). The data are presented as the means ± standard deviations (SD).

## 3. Results

### 3.1. Endogenous PIAS2 Restricts HCV Infection

**Figure 1:**
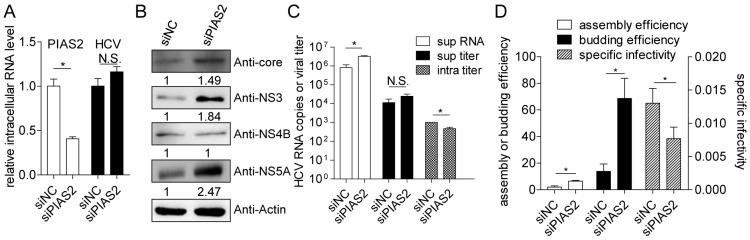
Endogenous protein inhibitor of activated STAT2 (PIAS2) functions as a restriction factor for hepatitis C virus (HCV) infection. Huh7 cells were transfected with a small interference RNA (siRNA) as indicated and then infected with J399EM at an multiple of infection (MOI) of 0.1 for 72 h. RNA and protein samples were collected to determine the relative intracellular PIAS2 mRNA levels and HCV RNA levels (**A**); and the HCV core, NS3, NS4B, and NS5A protein levels (**B**); (**C**) the HCV RNA level in the supernatant (sup RNA), viral titer in the supernatant (sup titer), and intracellular viral titer (intra titer) were determined; (**D**) the assembly efficiency (super RNA/intra RNA), budding efficiency (super titer/intra titer), and specific infectivity (super titer/super RNA) were calculated. siNC: scramble siRNA as negative control. N.S: not significant, * *p* < 0.05.

The function of endogenous PIAS2 in HCV infection was analyzed in the HCVcc system. Endogenous PIAS2 expression was knocked down using a specific siRNA (siPIAS2), and the HCV replication and propagation levels were monitored. The knockdown efficiency was indicated by the decreased intracellular PIAS2 mRNA level (Figure 1A). Although the intracellular HCV RNA levels were similar in the siPIAS2—and siNC (scramble siRNA as negative control)—transfected cells (Figure 1A), an obvious increase in the HCV core, NS3 and NS5A protein but not the NS4B protein levels was observed in the siPIAS2 transfected cells (Figure 1B). The HCV RNA level was approximately 4-fold higher in the supernatant from the siPIAS2 transfected cells than in the control cell supernatant, and a 2-fold decrease in the intracellular viral titer was observed (Figure 1C). The assembly efficiency, budding efficiency and specific infectivity were defined as the ratio of supernatant HCV RNA copies to intracellular HCV RNA copies, the ratio of the HCV titer in the cell supernatant to the HCV titer in the cells, and the ratio of HCV RNA copies to the HCV titer in the cell supernatant, respectively. The results shown in Figure 1D indicated that after siPIAS2 transfection, the assembly efficiency of HCV was upregulated by 3.3-fold, the budding efficiency was upregulated by approximately 4.9-fold, and the specific infectivity in the supernatant was slightly decreased. Clearly, reducing endogenous PIAS2 expression in Huh7 cells increased core, NS3 and NS5A protein expression and the HCV assembly and budding efficiencies, indicating a potential role for PIAS2 in HCV replication restriction.

### 3.2. Exogenously Expressed PIAS2 Inhibits HCV Replication

**Figure 2:**
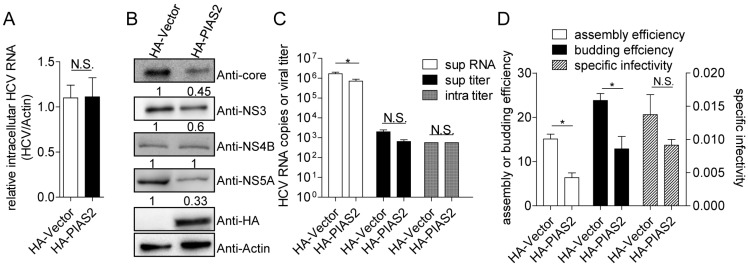
Exogenously expressed PIAS2 inhibits HCV replication. Huh7 cells were transfected with plasmids as indicated and then infected with J399EM at an MOI of 0.1 for 72 h. (**A**) The relative intracellular HCV RNA levels were determined by real-time RT-PCR; (**B**) the HCV core, NS3, NS4B, NS5A protein and HA-tagged PIAS2 expression levels are shown; (**C**) the HCV RNA level in the supernatant (super RNA), viral titer in the supernatant (super titer), and intracellular viral titer (intra titer) were determined; (**D**) the assembly efficiency, budding efficiency, and specific infectivity were calculated, * *p* < 0.05.

The inhibitory effect of PIAS2 on HCV replication was analyzed by comparing HCV replication and propagation in Huh7 cells transfected with the control vector or pHA-PIAS2. The intracellular HCV RNA level did not significantly change (Figure 2A), whereas the HCV core, NS3 and NS5A protein levels were markedly decreased in the PIAS2-overexpressing cells (Figure 2B). PIAS2 overexpression reduced the HCV RNA copy numbers in the supernatant by approximately 60% compared to the vector-transfected cells. No significant change in the viral titer was found in either the supernatant or the cells after PIAS2 overexpression (Figure 2C). The viral assembly efficiency was decreased by approximately 52% and the budding efficiency was reduced to 54%, although the specific infectivity was not affected (Figure 2D). Thus, PIAS2 overexpression reduced the HCV protein level, the assembly efficiency and the budding efficiency, indicating an inhibitory effect of PIAS2 on HCV replication.

### 3.3. PIAS2 Influences HCV Protein Expression in a Subgenomic Replicon System

**Figure 3:**
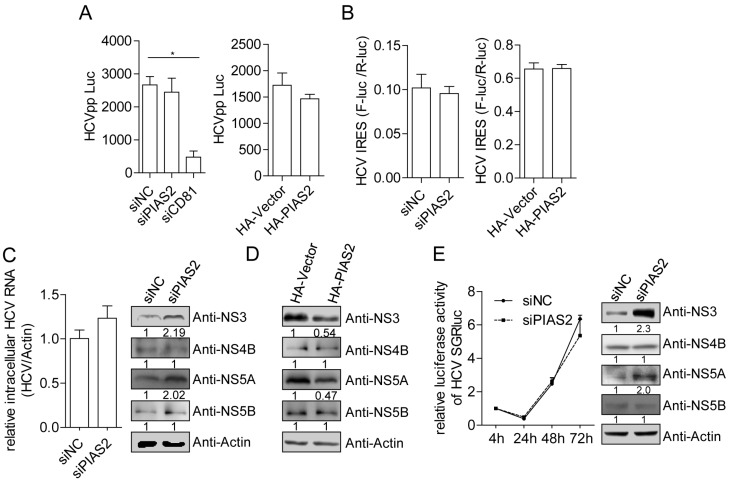
PIAS2 restricts HCV protein expression in the subgenomic replicon. (**A**) Huh7 cells were transfected with siRNA or plasmids as indicated for 48 h and then transduced with HCVpp. The luciferase activity was measured 48 h post-transduction; (**B**) Huh7 cells were transfected with the siRNA or plasmids as indicated and then transfected with the pHCV-internal ribosome entry site (IRES) reporter plasmid. The dual-luciferase assay was performed 48 h later. The IRES translation efficiency was determined by the ratio of firefly luciferase (F-Luc) activity to Renilla luciferase (R-Luc) activity; (**C**) Con1 cells were transfected with the indicated siRNAs. The HCV RNA and protein expression levels were detected at 72 h post-transfection. The numbers below each blot show the relative density of the blots normalized to actin; (**D**) Con1 cells were transfected with vector or pHA-PIAS2 plasmids and HCV NS3, NS4B, NS5A and NS5B protein expression were detected by western blotting; (**E**) Huh7 cells were electroporated with the JFH1-SGR-luc RNA (10 µg) together with the siNC or siPIAS2 (20 nM). Luciferase activity was measured at the indicated time points and normalized to the value obtained at 4 h post-electroporation. The HCV protein expression levels were detected at 72 h, * *p* < 0.05.

To determine whether PIAS2 was required for other steps in the HCV life cycle, we used the HCVpp transduction system, pHCV-IRES reporter plasmid and HCV subgenomic replicon system to explore the role of PIAS2 in HCV entry, IRES-dependent translation and RNA replication. Neither knockdown nor overexpression of PIAS2 affected the HCVpp entry or HCV IRES-dependent translation efficiency (Figure 3A,B), suggesting that PIAS2 did not influence the HCV entry and protein translation steps. Knockdown of PIAS2 expression in Con1 cells, which harbor the HCV genotype 1b subgenomic replicon, did not influence the HCV RNA level in the cells but significantly enhanced the NS3 and NS5A protein level (Figure 3C), while PIAS2 overexpression reduced NS3 and NS5A protein level (Figure 3D). Transient replication of HCV subgenomic RNA was subsequently tested in Huh7 cells electroporated with JFH1-SGR-luc RNA. Consistently, knockdown of PIAS2 expression did not change luciferase expression, which was translated under the control of the HCV IRES, but did enhance NS3 protein expression (Figure 3E); because the luciferase and HCV NS3 protein were translated from the same JFH1-SGR-luc RNA molecular plasmid under the control of different IRES structures and PIAS2 did not affect the translation efficiency of the HCV IRES, we concluded that PIAS2 had no effect on HCV RNA replication but instead modulated the stability of the HCV proteins.

### 3.4. PIAS2 Modulates HCV Core, NS3 and NS5A Protein Stability

**Figure 4:**
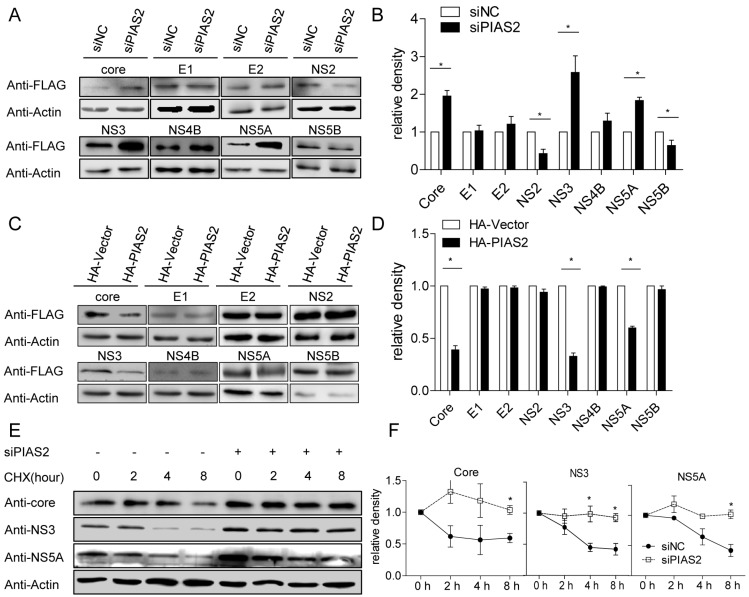
PIAS2 modulates the stability of the HCV core, NS3 and NS5A proteins. (**A**,**B**) 293T cells were transfected with siNC or siPIAS2 and then transfected with the indicated HCV protein expression plasmids. The expression levels of the HCV proteins were detected at 48 h post-transfection. The relative density of the blots from three independent experiments were analyzed by densitometry and shown in panel (**B**); (**C**,**D**) 293T cells were transfected with pHA-Vector or pHA-PIAS2, HCV protein expression plasmids, and pCer-SUMO1. The HCV protein expression levels were detected at 48 h post-transfection. The relative density of the blots from three independent experiments was analyzed by densitometry and shown in panel (**D**); (**E**) Huh7 cells were transfected with siNC or siPIAS2 and then infected with J399EM at an MOI of 0.1 for 72 h. The cells were treated with cycloheximide (CHX) (100 μg/mL), and protein samples were collected at the indicated time points to evaluate the protein expression levels as indicated; (**F**) the relative density of the blots from three independent experiments was analyzed by densitometry and normalized to actin. To show the degradation rate, the samples without CHX treatment in each group were set to 1, * *p* < 0.05.

Since PIAS2 changed the HCV protein expression levels in both the HCVcc and HCV subgenomic RNA replication systems, we tested whether PIAS2 affected the HCV protein levels expressed from individual plasmids. HCV protein expression from individual plasmids was evaluated in the absence or presence of PIAS2 in 293T cells. Compared with the siNC-transfected cells, the core, NS3 and NS5A protein expression levels were increased and the NS2 and NS5B expression levels were decreased in the siPIAS2-transfected group (Figure 4A,B). Strikingly, although PIAS2 overexpression did not change the HCV protein expression levels (Figure S1), PIAS2 expressed in tandem with SUMO1 significantly reduced the exogenous core, NS3 and NS5A protein expression levels (Figure 4C,D), suggesting a requirement for the PIAS2 E3 SUMO ligase function for the stabilization of the HCV core, NS3 and NS5A proteins.

To analyze the function of PIAS2 during HCV infection, we assessed the stabilities of the HCV core, NS3 and NS5A proteins in siNC- and siPIAS2-transfected cells. Cycloheximide (CHX) was added to the cell culture to block protein synthesis at 72 h post-HCVcc infection. The core, NS3 and NS5A protein levels were evaluated at the indicated time points post-CHX treatment. Knockdown of PIAS2 expression significantly increased the core, NS3 and NS5A protein levels (Figure 4E, compare lines 1 and 5). The core, NS3 and NS5A proteins were degraded gradually in the siNC-transfected cells, whereas this degradation was slowed down significantly in the siPIAS2-transfected cells (Figure 4E,F).

Overall, these results indicated that PIAS2 modulated the stability of the HCV core, NS3 and NS5A proteins, which probably required the E3 SUMO ligase function.

### 3.5. PIAS2 Influences HCV Protein Degradation through the Proteasome Pathway

**Figure 5:**
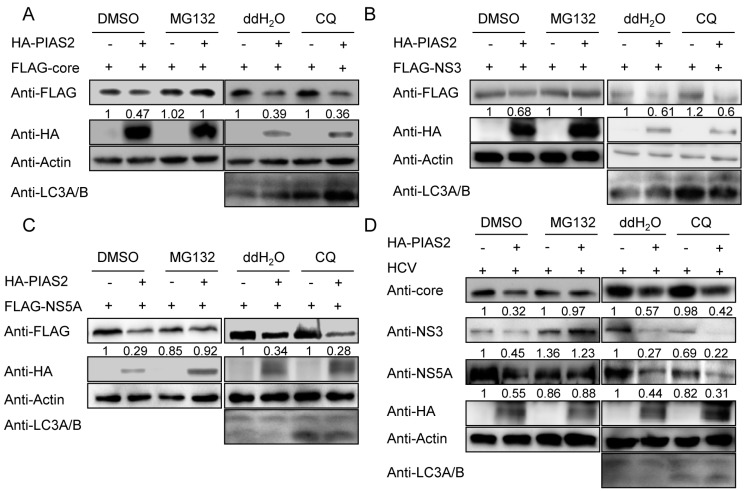
PIAS2 influences the degradation of HCV proteins through the proteasome pathway. (**A**–**C**) 293T cells were transfected with the indicated HCV protein expression plasmid, pCer-SUMO1 and pHA-Vector or pHA-PIAS2 and incubated for 48 h. Then, the cells were treated with MG-132 or chloroquine (CQ) for 4 h, and the protein expression levels were detected by western blotting; (**D**) Huh7 cells were transfected with either pHA-Vector or pHA-PIAS2 and infected with J399EM at an MOI of 0.1 for 72 h. After 4 h MG132 or chloroquine treatment, the expression levels of the HCV proteins were evaluated. The numbers below each blot show the relative density of the blots normalized to actin. DMSO: dimethyl sulfoxide.

The proteasome and lysosomes are the two known machineries responsible for protein degradation in eukaryotic cells. HCV proteins have been reported to be degraded through the ubiquitin-proteasome and/or autophagy-lysosome pathway. To analyze how PIAS2 modulated the stability of the HCV core, NS3 and NS5A proteins, we treated the cells with the proteasome inhibitor MG132 or the lysosome inhibitor chloroquine (CQ) and evaluated the extent of PIAS2-induced degradation of the HCV core, NS3 and NS5A proteins. MG132 but not CQ blocked the PIAS2-mediated decrease in the core, NS3 and NS5A protein level expressed from plasmids (Figure 5A–C). CQ treatment increased the LC3A/B level in cells, indicating that CQ could efficiently block the autophage-lysosome pathway in our system. Furthermore, PIAS2 lost its influence on the core, NS3 and NS5A expression levels in the presence of MG132 in the HCVcc system (Figure 5D). These results indicated that PIAS2 down-regulated core, NS3 and NS5A expression through the proteasome pathway.

### 3.6. The Influence of PIAS2 in HCV Protein Ubiquitination

**Figure 6:**
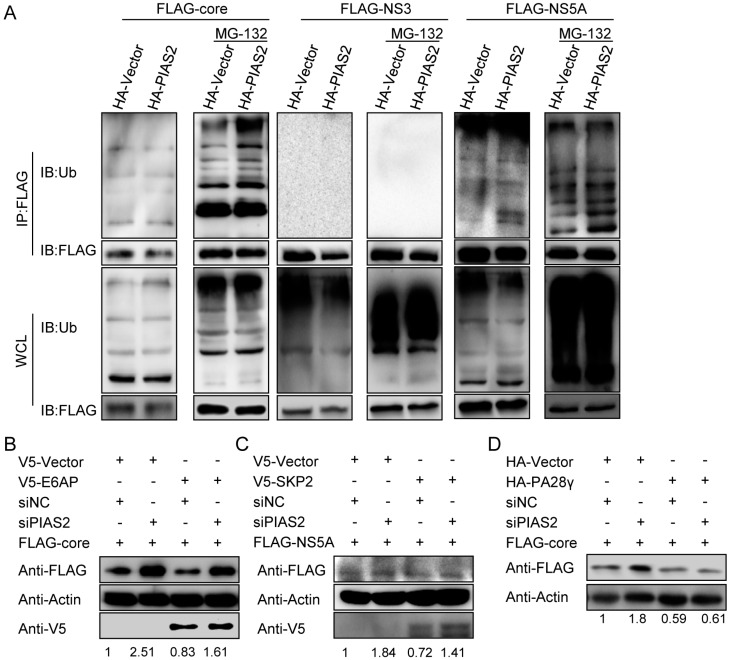
The influence of PIAS2 on HCV protein ubiquitination. (**A**) 293T cells were transfected with the indicated plasmids together with Ub and Cer-SUMO1 expression plasmids. MG132 treatment was performed 4 h before the total protein was extracted. Then, immunoprecipitation and western blotting were performed; (**B**–**D**) 293T cells were transfected with the siRNA and plasmids as indicated. Total protein was collected and detected by western blotting, WCL: whole cell lysate.

The ubiquitination levels of the core, NS3 and NS5A proteins were then evaluated. Because PIAS2 reduced HCV proteins expression only when SUMO1 was co-expressed, we transfected 293T cells with pHA-PIAS2 and HCV protein expression plasmid together with pUb and pCer-SUMO1. PIAS2 overexpression slightly enhanced the ubiquitination of the core and NS5A proteins in the presence of MG132, whereas no ubiquitination of NS3 was detected (Figure 6A). Because the E3 ligases E6AP and SKP2 were reported to enhance the ubiquitination and degradation of the core and NS5A, respectively, we tested whether PIAS2 influenced the functions of E6AP and SKP2. As reported, E6AP overexpression reduced the core protein level, and this effect was not influenced by PIAS2 knockdown; however, siPIAS2 still increased core protein expression when E6AP was overexpressed (Figure 6B). Similarly, SKP2-mediated NS5A degradation was not affected by PIAS2 silencing, and siPIAS2-enhanced NS5A expression was not affected by SKP2 overexpression (Figure 6C). Thus, PIAS2 probably mediated the degradation of the core, NS5A and NS3 proteins through a ubiquitin-independent proteasomal pathway. The ubiquitin-independent proteasomal degradation of the core protein was reported to be mediated by PA28γ. As shown in Figure 6D, the core protein expression level was not changed by PIAS2 silencing when PA28γ was overexpressed, suggesting that PIAS2 may mediate core degradation in a PA28γ-dependent manner.

### 3.7. PIAS2 Interacts with and Enhances SUMOylation of the Core Protein

**Figure 7:**
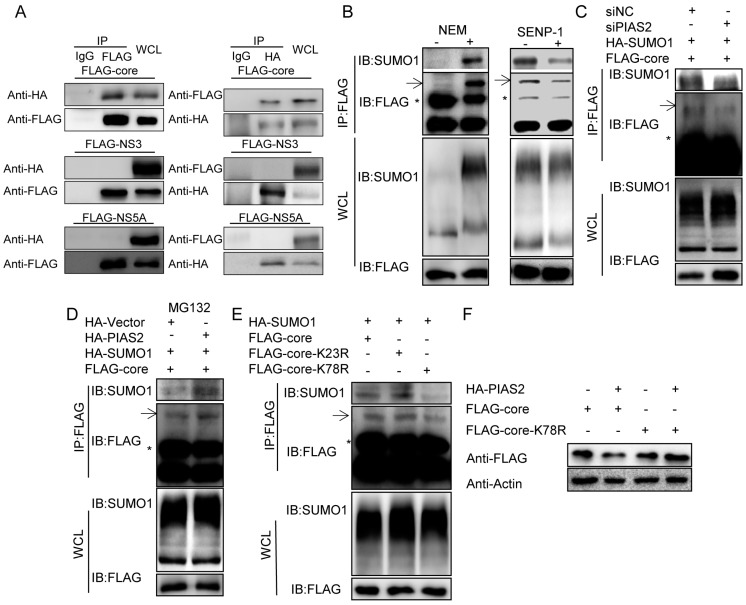
PIAS2 interacts with and enhances SUMOylation of the core protein. (**A**) 293T cells were transfected with pHA-PIAS2, HCV protein expression plasmids and pCer-SUMO1. Left panel, immunoprecipitation was performed with a FLAG or IgG antibody. Right panel, immunoprecipitation was performed with an HA or IgG antibody; (**B**) left panel, 293T cells were transfected with pHA-SUMO1 and pFLAG-core for 48 h. Total protein was collected with or without *N*-ethylmaleimide (NEM) in the lysis buffer, and immunoprecipitation was performed with a FLAG antibody. Right panel, 293T cells were transfected with pHA-SUMO1, pFLAG-core and p SUMO-sentrin specific protease (SENP-1) or pVector. Total proteins were collected using lysis buffer with NEM, and immunoprecipitation was performed with a FLAG antibody. The arrow shows the SUMOylated core protein band, and the asterisk indicates the location of the light chain; (**C**–**E**) 293T cells were transfected with siRNA and/or plasmids as indicated. Total protein was collected using lysis buffer with NEM. Immunoprecipitation followed by western blotting was performed with the indicated antibodies; (**F**) 293T cells were transfected with the plasmids as indicated, and the expression levels of the core protein were detected by Western blotting.

Because the E3 SUMO ligase function of PIAS2 was required for modulating the stability of the HCV core, NS3 and NS5A proteins, we asked whether PIAS2 interacted with and modified the SUMOylation of these proteins. A co-IP was performed to determine whether PIAS2 interacted with HCV proteins. When SUMO1 and PIAS2 were co-expressed together, PIAS2 was co-immunoprecipitated with the core protein but not NS3 nor NS5A using the flag antibody (Figure 7A left panel); and only core protein was co-immunoprecipitated with PIAS2 using the HA antibody (Figure 7A, right panel). Conversely, no interaction of the core proteins with PIAS2 was observed without SUMO1 overexpression (Figure S2), suggesting a requirement for SUMO1 in the PIAS2-core interaction and prompting us to investigate whether the core was SUMOylated by PIAS2. First, SUMOylation of the core protein was checked by adding the cysteine protease inhibitor NEM to the protein extraction buffer, which could stabilize SUMO1-modified proteins [40,41]. After accumulation by immunoprecipitation, a significant amount of core protein displayed a higher molecular weight species with a band shift consistent with a SUMOylated form of the protein (Figure 7B left, panel IB Flag). Indeed, SUMO1 was detected at this position (Figure 7B left, panel IB SUMO). When the SENP-1 protease, which can deconjugate SUMO from SUMOylated proteins, was co-transfected into the cells, the SUMOylated forms of the core proteins were reduced (Figure 7B right), further demonstrating that the core protein was modified by SUMO1. Then, the role of PIAS2 in core SUMOylation was analyzed. As shown in Figure 7C, the SUMOylated core was reduced and the total core protein was increased when PIAS2 was knocked down. PIAS2 overexpression increased the core SUMOylation in the presence of MG132 (Figure 7D), whereas this increase was not significant in the absence of MG132 treatment (Figure S3). These results suggested that the PIAS2 SUMOylated core was targeted for degradation via the proteasome pathway.

Possible SUMOylation sites in the core protein were analyzed using the SUMOylation predictor GSP-SUMO [42] by searching for the tetrapeptide consensus motif Ψ-K-x-D/E. The lysine amino acid at the possible SUMOylation site was mutated to arginine, and the SUMOylation of the core protein was evaluated. The SUMOylation level of core K78R was significantly reduced compared with the wild-type core protein (Figure 7E). Furthermore, PIAS2 did not decrease the core K78R protein expression level (Figure 7F). Thus, the lysine at amino acid position 78 of the core protein is a SUMOylation site mediated by PIAS2.

## 4. Discussion

In summary, PIAS2 was identified as a restriction factor for HCV replication. Utilizing different systems, including HCVcc, HCVpp, pHCV-IRES, JFH1-SGR-luc transient transfection and the HCV subgenomic replicon cell line, we determined that PIAS2 functioned in the HCV core, NS3 and NS5A protein expression at the post-translational level and thus influenced the assembly efficiency but not the other steps of the HCV life cycle, including viral entry, protein translation and RNA replication. The control of protein stability is specific to the core, NS3 and NS5A proteins, because the stability of the other HCV proteins exogenously expressed from plasmids was not controlled by PIAS2 (Figure 4C), and NS4B and NS5B expression in the HCVcc and HCV subgenomic replicons was not changed by PIAS2 silencing or overexpression (Figure 1B and Figure 3C–F).

PIAS2 mediated degradation of the core, NS3 and NS5A through a ubiquitin-independent proteasome pathway. Clearly, the SUMO1 E3 ligase activity of PIAS2 is required for the degradation of these three proteins, because PIAS2 only reduced the exogenous core, NS3 and NS5A protein expression levels when it was overexpressed with SUMO1 but not SUMO2/3 (Figure S4). However, the mechanisms underlying the PIAS2-mediated degradation of these proteins probably differ. In the literature, the stability of the core protein was degraded by E6AP-mediated ubiquitination and the PA28γ-mediated, ubiquitin-independent, nuclear proteasome pathway. Consistent with the previous literature [43,44], PIAS2 was mainly located in the nuclear fraction, whereas a small proportion of PIAS2 was found in the cytoplasmic fraction by immunofluorescence (IF) and nuclear-cytoplasmic fractionation (Figure S5). Considering that endogenous PIAS2 could not restrict the core protein level when PA28γ was overexpressed and that both PIAS2 and PA28γ were located mainly in the nuclear fraction, PIAS2 might mediate core degradation in a PA28γ-dependent manner in the nucleus. Moreover, an interaction between the PIAS2 and core proteins was detected by co-IP. We provide evidence that the core protein can be SUMOylated by PIAS2 at amino acid K78 and that this SUMOylation is required for PIAS2-mediated core degradation. The mechanisms underlying the PIAS2-mediated degradation of NS5A and NS3 are still unknown. Several factors, such as ISG12a [16], TRIM14 [45] and TRIM22 [46], were reported to influence the ubiquitination of NS5A, whereas PIAS2 did not significantly influence the NS5A ubiquitination level. Few reports have investigated the control of NS3 protein degradation, although HSP90 was suggested to stabilize NS3 [47]; however, HSP90 could not counteract PIAS2-mediated NS3 degradation (Figure S6). 

Our results indicated that PIAS2-mediated SUMOylation constrained HCV replication. Previously, Lee et al. reported that silencing of Ubc9, which is a key enzyme in the SUMO system, impaired HCV RNA replication [48]. HCV infection upregulated SUMO1 expression, and the depletion of SUMO1 impaired lipid droplet accumulation and HCV replication [49]. Both reports suggested the requirement of the SUMO system for HCV replication. Ubc9 is the only known SUMO E2 enzyme, whereas several SUMO E3 ligases have been identified. During the SUMOylation process, E1 and E2 are sufficient to finish the attachment of SUMO to proteins, although the E1, E2 and E3 pathway is more efficient. SUMO E3 ligases may play a role in regulating substrate selection [50]. Thus, depletion of SUMO1 or silencing of Ubc9 could impair the SUMOylation machinery and significantly influence proteostasis. In contrast, silencing of PIAS2 may only affect the SUMOylation of a group of specific proteins, which may explain the discrepancy between Ubc9 and PIAS2-mediated SUMOylation in HCV replication. Future studies to identify the specific targets of PIAS2 and further explore their functions in HCV replication will be interesting.

## Supplementary Materials

The following are available online at www.mdpi.com/1999-4915/9/10/285/s1.
