# Supplementary material for: Protein Inhibitor of Activated STAT2 Restricts HCV Replication by Modulating Viral Proteins Degradation
Source: Viruses. 2017 Sep 30;9(10):285. doi: 10.3390/v9100285 (PMC5691636; doi:10.3390/v9100285)
Supplement: Supplementary file 1 [file viruses-09-00285-s001.pdf]

Supplementary Result

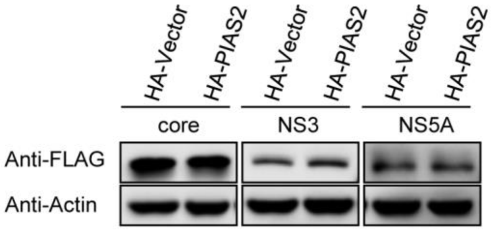

Figure S1. Protein inhibitor of activated STAT2 (PIAS2) alone didn't influence the expression level of Hepatitis C virus (HCV) core, NS3 or NS5A. 293T cells were transfected with pHA-Vector or pHA-PIAS2, HCV core, NS3 or NS5A expression plasmids. Total protein samples were collected and the expression levels of core, NS3 and NS5A were detected by western blotting.

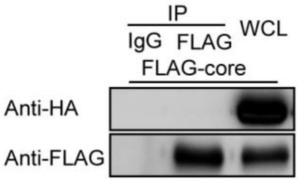

Figure S2. HCV core could not interact with PIAS2 without overexpression SUMO1. 293T cells were over-expressed with pHA-PIAS2, pFLAG-core. Co-immunoprecipitation was performed with FLAG or IgG antibody.

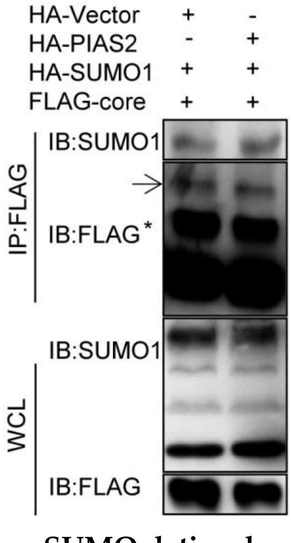

Figure S3. The changes in core SUMOylation level by PIAS2 was not observed PIAS2 without MG132 treatment. 293T cells were transfected with plasmids as indicated. Total proteins were collected by lysis Buffer with *N*-ethylmaleimide (NEM). Immunoprecipitation followed by western blotting were performed with indicated antibodies.

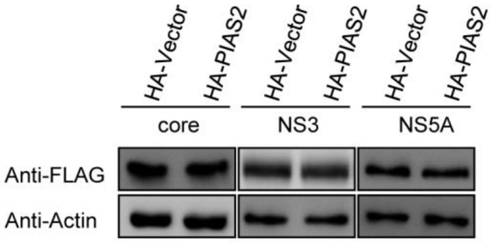

Figure S4. In the presence of SUMO2/3, PIAS2 didn't influence the expression level of HCV core, NS3 or NS5A. 293T cells were transfected with pHA-Vector or pHA-PIAS2, HCV core, NS3 or NS5A expression plasmids together with pCer-SUMO2/3. Total protein samples were collected and the expression levels of core, NS3 and NS5A were detected by western blotting.

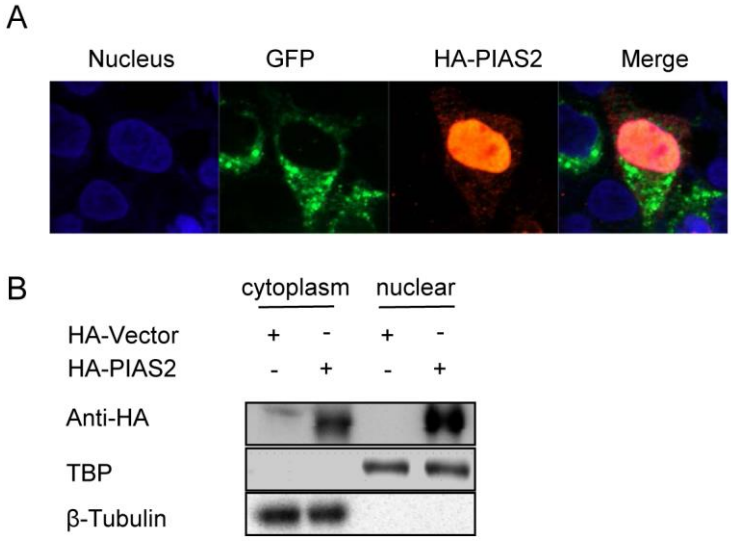

Figure S5. PIAS2 mainly locates in the nucleus. (A) Huh7 cells were transfected with pHA-PIAS2 and then infected with J399EM at an MOI of 0.1. At 72 hours post-infection, the cells were fixed with 4% paraformaldehyde and washed three times with PBS. The fixed cells were blocked in PBS containing 1% NGS at 37°C for 1 h and incubated with the primary antibody (anti-HA monoclonal antibody) for 1 h at 37°C. Subsequently, the cells were washed three times with PBS and incubated with a secondary antibody [Alexa-conjugated donkey anti-mouse/rabbit IgG antibody (Invitrogen)] for 1 h at 37°C. The cells were counterstained with DAPI, washed with PBS and examined under a confocal laser microscope. The localization of NS5A was shown by EGFP and immunofluorescence was performed to show the location of PIAS2. (B) Huh7 cells were transfected with pHA-Vector or pHA-PIAS2. Nuclear and cytosol proteins were separated with nuclear/cytosol fraction kit and the subcellular fractions were detected by western blotting.

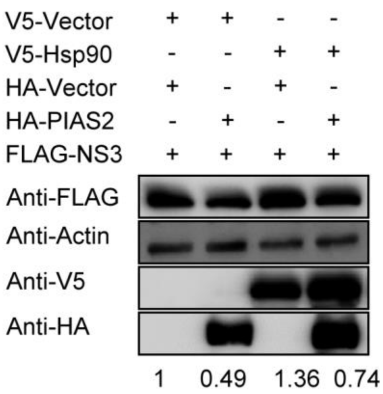

Figure S6. Heat shock protein 90 (HSP90) did not interfere PIAS2 mediates NS3 degradation. 293T cells were transfected with plasmids as indicated. The proteins expression was detected by western blotting.
